# An Emerging Pulmonary Haemorrhagic Syndrome in Dogs: Similar to the Human Leptospiral Pulmonary Haemorrhagic Syndrome?

**DOI:** 10.4061/2010/928541

**Published:** 2010-12-27

**Authors:** R. Klopfleisch, B. Kohn, S. Plog, C. Weingart, K. Nöckler, A. Mayer-Scholl, A. D. Gruber

**Affiliations:** ^1^Institute of Veterinary Pathology, Department of Veterinary Medicine, Freie Universität Berlin, Robert-von-Ostertag-Straße 15, 14163 Berlin, Germany; ^2^Small Animal Clinic, Department of Veterinary Medicine, Freie Universität Berlin, Oertzenweg 19 b, 14163 Berlin, Germany; ^3^Department of Molecular Diagnostics, Federal Institute for Risk Assessment, Diedersdorfer Weg 1, 14191 Berlin, Germany

## Abstract

Severe pulmonary haemorrhage is a rare necropsy finding in dogs but the leptospiral pulmonary haemorrhagic syndrome (LPHS) is a well recognized disease in humans. Here we report a pulmonary haemorrhagic syndrome in dogs that closely resembles the human disease. All 15 dogs had massive, pulmonary haemorrhage affecting all lung lobes while haemorrhage in other organs was minimal. Histologically, pulmonary lesions were characterized by acute, alveolar haemorrhage without identifiable vascular lesions. Seven dogs had mild alveolar wall necrosis with hyaline membranes and minimal intraalveolar fibrin. In addition, eight dogs had acute renal tubular necrosis. Six dogs had a clinical diagnosis of leptospirosis based on renal and hepatic failure, positive microscopic agglutination test (MAT) and/or positive blood/urine *Leptospira*-specific PCR. *Leptospira* could not be cultured post mortem from the lungs or kidneys. However, *Leptospira*-specific PCR was positive in lung, liver or kidneys of three dogs. In summary, a novel pulmonary haemorrhagic syndrome was identified in dogs but the mechanism of the massive pulmonary erythrocyte extravasation remains elusive. The lack of a consistent post mortem identification of *Leptospira* spp. in dogs with pulmonary haemorrhage raise questions as to whether additional factors besides *Leptospira* may cause this as yet unrecognized entity in dogs.

## 1. Introduction

Severe pulmonary haemorrhage is a rare necropsy finding in dogs and may be caused by pulmonary-renal syndrome, heartworm disease, thrombocytopenia, or coagulopathy, it but has not been reported in natural leptospirosis infection [1–4]. However, in an experimental model of canine leptospirosis, acute pulmonary haemorrhage was found in some animals in addition to the predominant and common findings of nephritis and renal tubular epithelial degeneration in some animals [5]. In clinical and radiographic evidence of pulmonary manifestation in dogs, natural *Leptospira*-infection has been reported rarely [6–8]. 

Human infections with pathogenic *Leptospira *serovars are often associated with the leptospiral pulmonary haemorrhagic syndrome (LPHS), a well-recognized entity in man [3, 9, 10]. LPHS is associated with fatality rates of >50% and in certain settings has replaced Weil's disease with spleen enlargement, jaundice, and renal failure as the most common cause of death among human leptospirosis patients [11–13]. Clinical-pathological findings in humans with LPHS include acute respiratory distress syndrome (ARDS) with hemoptysis, focal pulmonary haemorrhage, and multiorgan failure [14]. The microscopic agglutination test (MAT) is considered the gold standard for laboratory diagnosis of LPHS in humans and is based on detection of *Leptospira*-specific immunoglobulin subtype M (IgM, titre >1 : 400 in endemic regions) in the acute stage of the disease [15]. Cultivation is often difficult and not sufficiently sensitive due to the rapid decay of the leptospiral organisms [15]. 

The pathogenesis of pulmonary injury in LPHS is poorly understood, but vascular damage due to a leptospiral toxin, immunologic mechanisms, or a disseminated intravascular coagulopathy have been proposed as potential pathologic mechanisms [14, 16–18]. In addition, leptospiral organisms are rarely isolated, and leptospiral antigen rarely detected in the lungs or other organs [19]. A direct relationship between the antigen and lesions, therefore, is not established in LPHS. Vascular necrosis or exposure of subendothelial antigen were not observed in the lungs of affected patients, although endothelial cells in these patients were ultrastructurally swollen and contained increased amounts of pinocytotic vesicles and giant dense bodies in their cytoplasm [19].

Between 2006 and 2010, an increasing number of dogs suspicious for leptospirosis were treated at the Small Animal Clinic of the Freie Universität Berlin. More than two-third of these dogs had clinical and radiological pulmonary manifestation in addition to renal and hepatic insufficiency [20]. Clinical symptoms, MAT (serum), and *Leptospira*-specific PCR of urine or blood supported the tentative diagnosis of an unusual form of leptospirosis in these dogs. Interestingly, leptospirosis is of increasing importance and prevalence in the rural areas of Berlin, where the population of urban wild boars is growing dramatically, and these wild boars have recently been shown to represent a significant reservoir for several *Leptospira* serovars [21].

In this report, we describe the macroscopical and histopathological lesions in 15 of these dogs. Due to the similarities between the canine disease and the human LPHS, we also tested the hypothesis that *Leptospira sp.* may be the etiologic cause of the disease.

## 2. Material and Methods

### 2.1. Clinical History

Between 2006 and 2010, 43 dogs suspicious for a *Leptospira* infection developed respiratory distress and a focal or generalized interstitial/alveolar lung pattern on thoracic radiographs [20]. All dogs also had acute renal failure and/or hepatopathy that could not be attributed to other causes. 15 of these dogs, all from urban areas in Berlin, were submitted for full necropsy, and tissue samples were taken 24 to 72 hours postmortem (Table 1). 12 of the dogs were euthanized or died due to severe pulmonary distress and hemoptysis, while three of the animals were mainly euthanized due to severe renal failure. Six of the dogs (dogs nos. 1–6) were submitted with a clinical diagnosis of leptospirosis due to detection of leptospiral DNA in blood or urine (dogs nos 1–3, 5-6) or positive leptospiral MAT (dog no. 4, MAT >1 : 800, Serovar grippotyphosa, see [20]). Tissue samples of major organ systems, including the respiratory, urogenital, and gastrointestinal tract of all animals were submitted for bacteriology or routinely fixed in formalin, wax embedded, and stained with hematoxylin and eosin, Levaditi stain for detection of leptospiral organisms in kidney and lung, and Periodic acid-Schiff- (PAS-) reaction and phosphotungstic acid haematoxylin- (PTAH-) staining for detection of intravascular fibrin thrombi. Tissue samples of a confirmed case of renal leptospirosis were used as positive control for the Levaditi stain. Tissues of 3 animals were submitted for toxicological analysis of coumarin derivatives. Additionally, lung tissue samples from dogs nos. 4–9 were snap frozen and stored at −80°C for PCR detection of leptospiral DNA.

### 2.2. Immunohistochemistry and Immunofluorescence

*Leptospira interrogans*-specific rabbit antisera against serogroups Canicola, Icterohaemorrhagiae, Grippotyphosa, and Pomona (Dr. Nöckler, Federal Institute for Risk Assessment) were used for detection of leptospiral antigen in fresh-frozen and paraffin-embedded tissue lung and kidney sections using immunofluorescence test or ABC-method. In brief, all four antisera were pooled at equal amounts and diluted 1 : 25 in Tris-buffered saline (TBS, 50 mM, pH 7.6) and incubated at 4°C overnight after a blocking step with 50% goat serum in TBS (30 min at room temperature). For paraffin sections, goat antirabbit IgG (pab, 1 in 200; Vector, England, BA1000) and for immunofluorescence testing a Dylight 549-conjugated secondary antirabbit antibody (pab, 1 : 200, Dianova, Germany) were used as secondary antibodies (60 min at room temperature). Diaminobenzidine tetrahydrochloride (Sigma Aldrich) was used as chromogen and slides were counterstained with haematoxylin (Merck GmbH, Darmstadt, Germany). A Levaditi-stain-positive canine kidney was used as positive control, and lung sections of dogs without the pulmonary haemorrhage syndrome were similarly incubated and used as negative controls. No unspecific staining was detected in any tissue examined.

### 2.3. Bacteriology and PCR

Tissue samples of major organ systems, including the respiratory, urogenital, and gastrointestinal tract of all animals were submitted for cultivation in EMJH medium and routine bacteriology [15]. Cultures were maintained for 8 weeks.

Complete DNA was isolated using Nucleospin Tissue Kit (Macherey-Nagel, Düeren, Germany) according to the manufacturer instructions. For detection of *Leptospira* DNA, two standard primer pairs were used [22]. These primer pairs are named G1/G2 and B64I/B64II and are known to amplify a 285 bp and 585 bp product, respectively. Total genomic DNA was used as template in each reaction to amplify leptospiral DNA in a conventional PCR protocol according to [22]. Each tissue sample was tested at least two times. DNA from *L. interrogans *serovar Icterohaemorrhagiae (*sec*Y gene) und *L. kirschneri* serovar Grippotyphosa (*fla*B gene) was used as positive control in all PCR reactions. Furthermore, lung and kidney tissue of three dogs without pulmonary haemorrhage, but other neoplastic diseases, were included as negative controls.

## 3. Results

### 3.1. Necropsy Findings

**Figure 1:**
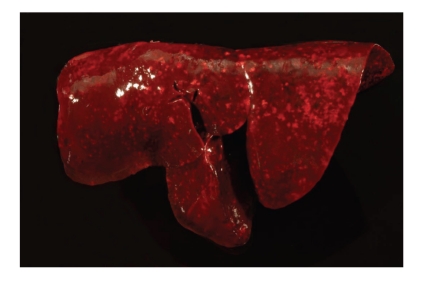
Lung, dog no. 6. Severe, acute, diffuse pulmonary haemorrhage.

Necropsy revealed severe, diffuse, acute pulmonary haemorrhage, and alveolar oedema affecting all lung lobes of all animals (Table 2). Lungs were wet and heavy and had a dark-red colour (Figure 1). Abundant noncoagulated blood was found on cut surfaces, and major airways were filled with abundant dark-red, foamy fluid. Pulmonary and mediastinal lymph nodes were also dark red with acute haemorrhage and blood resorption. Five of 15 animals had minimal petechiation on the mucosa of the urinary bladder. Four of 15 animals had mild to moderate jaundice, and three of 15 dogs had minimal to mild, acute haemorrhage in the gall bladder. Minimal to mild melena characterized by dark-red to black faeces was present in 4 of 15 animals. The gastric mucosa, kidneys, stomach, intestinal tract, and livers of all animals were unremarkable at necropsy. 

### 3.2. Histopathology

**Figure 2:**
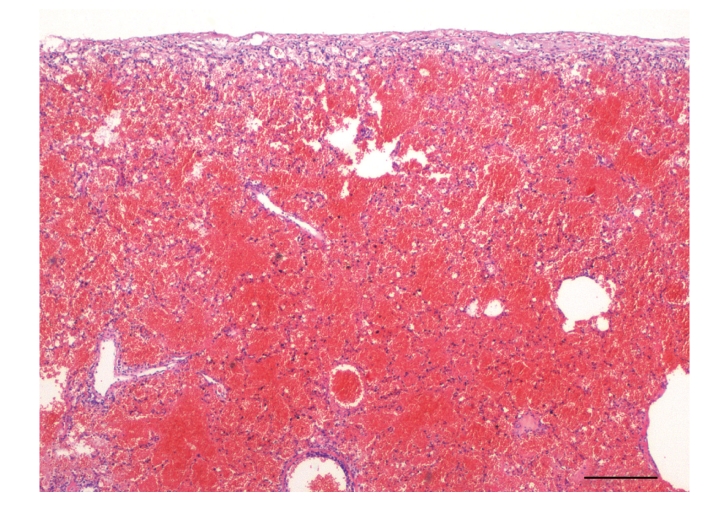
Lung, dog no. 7. Alveoli were diffusely and markedly filled with mostly uncoagulated blood in all dogs. HE stain.

**Figure 3:**
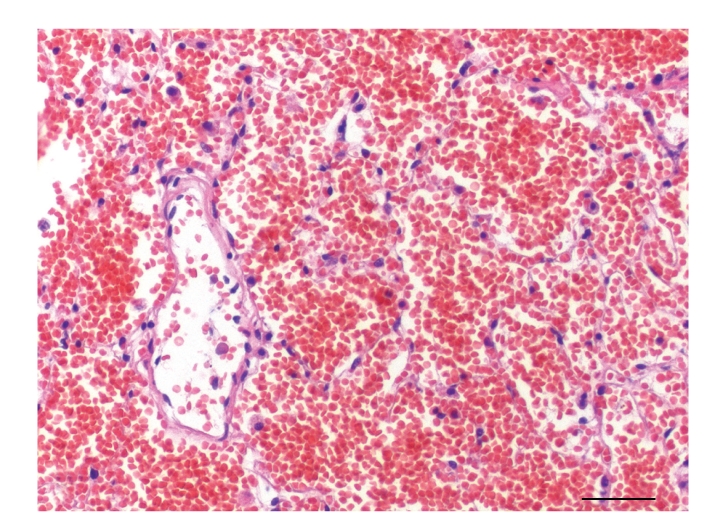
Lung, dog no. 7. Alveoli of more than 50% of the dogs contained numerous erythrocytes without signs of coagulation, alveolar hyaline membranes, or pneumocyte necrosis. HE stain.

**Figure 4:**
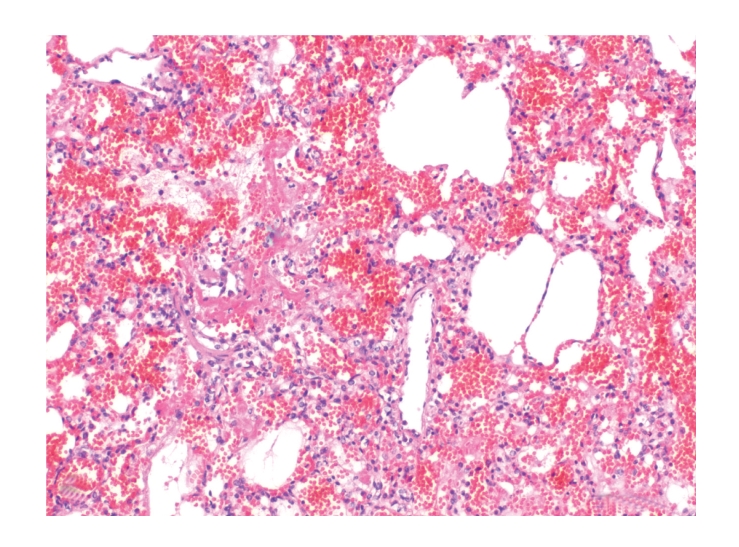
Lung, dog no. 9. Seven of 15 dogs had, however, intraalveolar fibrin deposition, formation of hyaline membranes, and mild pneumocyte necrosis. HE stain.

**Figure 5:**
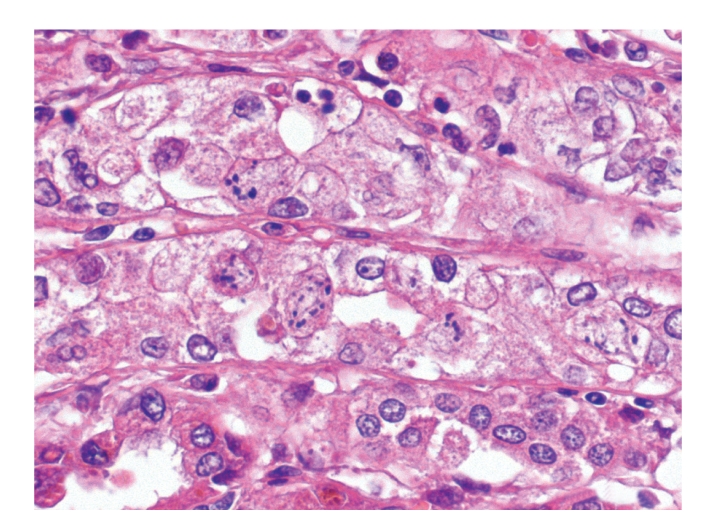
Kidney, dog no. 10. More than half of the dogs had acute, renal tubular necrosis. HE stain.

Histologically, pulmonary alveoli of all lungs were filled with erythrocytes, mostly without extravascular fibrin (Figures 2 and 3). Seven of 15 dogs had mild pneumocyte necrosis and occasionally small amounts of intraalveolar fibrin deposition and hyaline membranes (Figure 4, Table 2). Inflammatory cell infiltrates were not observed in any of the dogs. Erythrophagocytosis was rare or absent in all dogs. All dogs had unremarkable blood vessels of all calibres or rather no signs of pulmonary vessel wall disintegration or thrombosis. The mediastinal lymph node also had diffuse acute haemorrhage. Eight of 15 dogs had mild to severe, acute, diffuse renal proximal tubular epithelial necrosis with first evidence of tubular regeneration (Figure 5). Furthermore, four of 15 animals had mild, acute, centrolobular hepatocellular degeneration. 

PTAH and PAS stain failed to identify fibrin thrombi in vessels of all calibres and all organs analyzed. Furthermore, Levaditi stain failed to identify *Spirochaete*-like structures in renal tubular epithelium or lungs.

### 3.3. Immunohistochemistry and Immunofluorescence

Immunohistochemistry on paraffin sections as well as immunofluorescence testing failed to identify leptospiral antigen in lung or kidney of all dogs.

### 3.4. Bacterial Cultivation and PCR Findings

**Figure 6:**
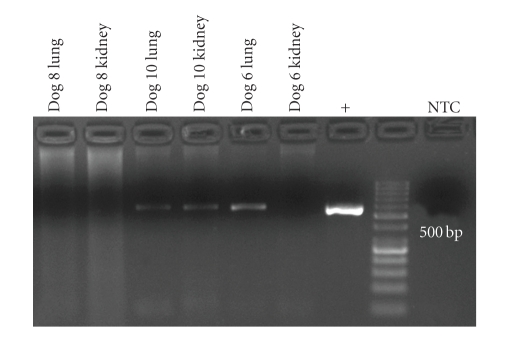
Detection of leptospiral-specific *fla*B gene DNA by PCR and gel electrophoresis in lungs and kidneys of dogs with pulmonary haemorrhagic syndrome. Specific PCR products were detected in lungs of dogs nos. 6 and 10, and kidney of dog no. 10.

**Figure 7:**
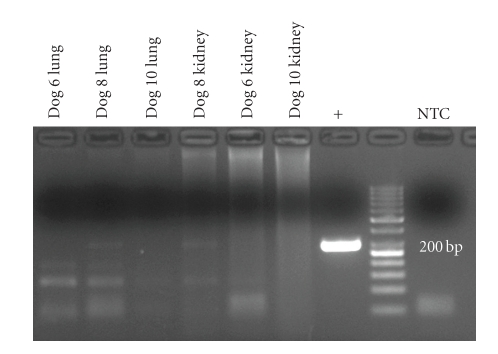
Detection of leptospiral-specific *sec*Y gene DNA by PCR and gel electrophoresis in lungs and kidneys of dogs with pulmonary haemorrhagic syndrome. Specific, but very weak, products were only detected in lungs and kidneys of dog no. 8. +: positive control, NTC: no template control.

Bacteriological examination of all lung and kidney samples failed to identify *Leptospira *spp. or other obligatory pathogenic microorganisms under aerobic or anaerobic conditions using EMJH medium and standard diagnostic procedures. By means of PCR, a *Leptospira*-DNA amplicon targeting the *fla*B gene (563 bp) was detected in the lungs of dogs Nos. 6 and 10 and in the kidney of dog No. 10 (Figure 6). Furthermore, the DNA-specific product for *sec*Y gene (285 bp) was present in lung and kidney of dog No. 8 (Figure 7). Lungs and kidneys of the three dogs without pulmonary haemorrhage were negative in all assays.

## 4. Discussion

In the present report, we describe a pulmonary haemorrhage syndrome in dogs and hypothesize whether this syndrome might be associated with a leptospiral infection similar to the human leptospiral pulmonary haemorrhagic syndrome (LPHS). 

Clinically, 6 of the dogs had a diagnosis of acute leptospirosis based on serological and PCR findings. Postmortem PCR detected leptospiral DNA in the lung, kidney, and liver of three dogs. Nevertheless, all other methods, including immunohistochemistry, immunofluorescence test, and Levaditi stain failed to identify leptospiral organisms in all dogs. Furthermore, Postmortem bacterial culturing did not isolate leptospiral organisms from the dogs. One explanation for the failure to cultivate leptospiral organisms even in PCR-positive dogs might be the antibiotic treatment of some of the dogs (personal communication with referring veterinarians). The low sensitivity of bacteriological and PCR-based methods to detect leptospiral organisms is well known and a major problem in the diagnosis of leptospirosis in general [23]. PCR is known as the most sensitive technique to identify leptospiral organisms in tissue [24]. However, only 30% of confirmed cases of human leptospirosis were identified with PCR [24], and a similar percentage of cases (3/15) have been identified by PCR in the present study and may have contributed to the differences between clinical and postmortem diagnosis of leptospirosis in the dogs. 

Nevertheless, the failure of postmortem identification of cultivatable leptospiral organisms and the lack of amplifiable leptospiral DNA may also point towards a very low bacterial load in the dogs. Similar to the human disease, pulmonary lesions may, therefore, not be caused by an infection of pulmonary structures, but rather induced by a bacterial toxin or an immunopathologic mechanism induced by bacterial components [14, 17, 18]. 

Alternatively, the clinical detection of leptospiral DNA in blood and urine and the increase of MAT titters in some dogs could represent an incidental finding of and is unrelated to the aetiology of the pulmonary lesions [20]. Subclinical, latent leptospirosis in dogs has regularly been reported and can also be observed in unsteady vaccinated animals [15, 25]. 

Obviously, the difficulties to consistently identify leptospiral organisms raises questions as to whether other factors may be involved in the pathogenesis of the disease. Other bacterial organisms cannot finally be excluded at this point, but failure to detect such organisms with routine bacterial culturing methods decreases the probability of a nonleptospiral bacterial causative agent. Viral infections may be a potential aetiology for the pulmonary haemorrhage, but known viruses such as highly pathogenic influenza H5N1 do not cause similar lesion in dogs or other carnivores [26–28]. Thus, further extensive, explorative virological testing is necessary to exclude a viral infection. In any case, the inconsistent identification of leptospiral organisms in the affected dogs does not unequivocally support the hypotheses of a leptospira-associated haemorrhagic syndrome in dogs similar to the human LPHS. However, leptospiral organisms, DNA, or antigen are also only inconsistently identified in the human cases of LPHS, and, in humans, the etiologic diagnosis is mostly based on serology data alone [19]. 

The main necropsy finding and cause of death in all dogs was severe, acute, pulmonary haemorrhage. It is remarkable that the pulmonary vascular system is almost exclusively affected by the disease since disseminated haemorrhage or microthrombi were not observed in the lungs or other organs. Only few animals had petechiation in the mucosa of the urinary bladder or melena. The latter was attributed to swallowing of coughed-up blood, since oesophageal and gastric mucosa was unremarkable. Surprisingly, alveolar wall necrosis or vessel wall disintegration was only detected in 50% of the affected lungs. The location of erythrocyte extravascularisation within the lung, therefore, remains elusive. The striking tropism for the lung and the lack of evidence of a general haemorrhagic diathesis suggest that pulmonary microvessels may represent a highly selective target of the mechanism involved, as hypothesized in human LPHS [19]. Generally, this assumption is supported by new findings in the human leptospiral disease, that suggest the lung as a common target of diseases related to systemic inflammation [29]. Although not identified yet, a bacterial cytotoxin from certain leptospiral isolates or possibly other bacteria may selectively target pulmonary endothelial cells. However, no vascular changes or microvascular thrombosis were noted in the 15 dogs. Affected vessels may thus be restricted to a rare endothelial subset particularly vulnerable to an alleged, and so far undescribed, noxa. Alveolar septal deposition of immunoglobulin and complement have been shown to involved in pulmonary haemorrhage in a guinea pig model of severe pulmonary leptospirosis [30]. However, histologically detectable amounts of antibody complexes were not observed in the present cases, but further immunohistochemical studies are necessary to finally evaluate this hypothesis.

Importantly, the pulmonary lesions were diffusely distributed indicating that whatever cell type or target structure may be linked to the cause of the haemorrhages, it is homogeneously distributed throughout the lungs. 

A disorder of the primary or secondary hemostasis as the sole cause for pulmonary haemorrhage seems unlikely since extensive haemorrhages were almost exclusively observed in the lungs. For instance, coumarin intoxication was excluded in 3 dogs by toxicological analysis; moreover, coagulation screening tests (activated partial thromboplastin time, prothrombin time) performed in several dogs made coumarin intoxication unlikely (data not shown). The role of thrombocytopenia in this disease also remains elusive. Low platelet counts are a common finding in canine leptospirosis but are generally, and in the specific context of leptospiral diseases, not associated with severe pulmonary haemorrhage [31]. The mechanism of pulmonary haemorrhage in the dogs, therefore, remains to be established, similar to human LPHS [14, 17, 18]. 

The acute liver lesions seen in some dogs are most likely associated with hypoxia due to respiratory distress, and the same may be true for the renal tubular degeneration. Nevertheless, renal and hepatic lesions may also be caused by leptospiral organisms, although levaditi stain and immunohistochemistry failed to identify leptospiral organisms in affected livers and kidneys.

In summary, we describe a new pulmonary haemorrhagic syndrome in dogs with striking clinical and pathological similarities to LPHS in man. However, leptospiral organisms, as the suspected cause, were not consistently identified with any of the diagnostic methods applied. The cause of the emerging pulmonary haemorrhage syndrome in dogs, therefore, remains unclear. Further studies are needed to finally determine whether leptospiral organisms or other causes are associated with the canine pulmonary haemorrhage syndrome.

## Figures and Tables

**Table 1:** Clinical and laboratory findings, PCR and immunohistochemical detection of Leptospira spp. in 15 dogs with acute pulmonary haemorrhage syndrome.

Animal	Azotemia/increased hepatic enzymes	Thrombocytopenia	MAT [20]	*Leptospira* detection by PCR [20]	*Leptospira* detection by IHC (lung, liver, kidney)
Animals with a clinical diagnosis of leptospirosis [20]					
1	+/+	+	−	Urine	−
2	+/+	+	−	Blood	−
3	+/+	+	−	Urine, blood	−
4	+/+	+	+	-	−
5	+/+	+	−	Urine, blood	−
6	+/+	+	−	Urine, blood, lung	−

Animals without a clinical diagnosis of leptospirosis					
7	+/+	+	−	−	−
8	n.t.	n.t.	n.t.	Lung, kidney	−
9	n.t.	n.t.	n.t.	−	−
10	n.t.	n.t.	n.t.	Lung, kidney	−
11	n.t.	n.t.	n.t.	−	−
12	n.t.	n.t.	n.t.	−	−
13	n.t.	n.t.	n.t.	−	−
14	n.t.	n.t.	n.t.	−	−
15	n.t.	n.t.	n.t.	−	−

n.t.: Not tested.

MAT titers of >1 : 800 were considered positive.

**Table 2:** Necropsy findings in dogs with pulmonary haemorrhage syndrome in the order of their frequency.

Diagnosis	Number of affected animals
Pulmonary haemorrhage	15/15
Renal tubular necrosis	8/15
Alveolar hyaline membranes	7/15
Pneumocyte necrosis	7/15
Petechiation, extrapulmonary haemorrhage, melena	5/15
Centrilobular hepatocellular necrosis	4/15
Jaundice	4/15
